# Life-threatening Gastrointestinal Bleeding Secondary to Kaposi’s Sarcoma of the Duodenum

**DOI:** 10.7759/cureus.7322

**Published:** 2020-03-19

**Authors:** Hanan Ibrahim, Mouhanna Abu Ghanimeh, Zaid I Al-Saheli, Sandra Naffouj

**Affiliations:** 1 Internal Medicine, Henry Ford Hospital, Detroit, USA; 2 Internal Medicine, Henry Ford Health System, Detroit, USA

**Keywords:** hiv, kaposi's sarcoma, aids, gastrointestinal bleeding

## Abstract

The use of antiretroviral therapy has decreased the incidence of human immunodeficiency virus (HIV) and acquired immunodeficiency syndrome (AIDS) complications. However, Kaposi’s sarcoma (KS) is not uncommon. KS can involve any organ, including the gastrointestinal tract. The disease usually remains asymptomatic, but hemorrhages have been reported due to the hypervascular nature of the lesions. We report a case of a newly diagnosed HIV-infected patient, who presented with upper gastrointestinal bleeding. His bleeding had become life-threatening after an adequate endoscopic sampling of the lesions to the extent where he was transferred to the intensive care unit and required multiple units of blood product transfusion and a selective embolization by interventional radiology to achieve hemostasis.

## Introduction

Kaposi’s sarcoma (KS) is a known complication of acquired immunodeficiency syndrome (AIDS) [1]. Any body organ can be involved, including the gastrointestinal tract. The manifestation of gastrointestinal involvement varies; the disease can be asymptomatic or it can cause other nonspecific gastrointestinal related symptoms, like nausea, weight loss, malabsorption, and gastrointestinal bleeding. Life-threatening gastrointestinal bleeding related to KS is rare and has been described only in case reports [2]. Diagnosis of KS requires the pathological identification of the spindled endothelial cells, and thus, adequate sampling is warranted [3].

## Case presentation

A 58-year-old bisexual male who has been recently diagnosed with human immunodeficiency virus (HIV) infection a few months back, presented to the clinic with progressive shortness of breath for two weeks. Over the last few months, he reported palpitations, lightheadedness, 50 pounds of unintentional weight loss, and non-tender, non-pruritic purple lesions on the body and scalp. He denied fever, chills or abdominal pain. On physical examination, he was afebrile, tachycardic (104 beats/minute) and hypotensive (93/58 mmHg). Precordial and chest auscultation revealed normal S1 and S2 and decreased breath sounds due to the patient’s dyspnea. His complete blood count showed a hemoglobin of 5.8 g/dl, white blood cell count of 7 X 103/mm3. His CD4 count was 120 cells/mm3 and the viral load was 194,373 copies/ml. He was started on antiretroviral therapy recently. The decision was made to admit the patient for further evaluation.

**Figure 1:**
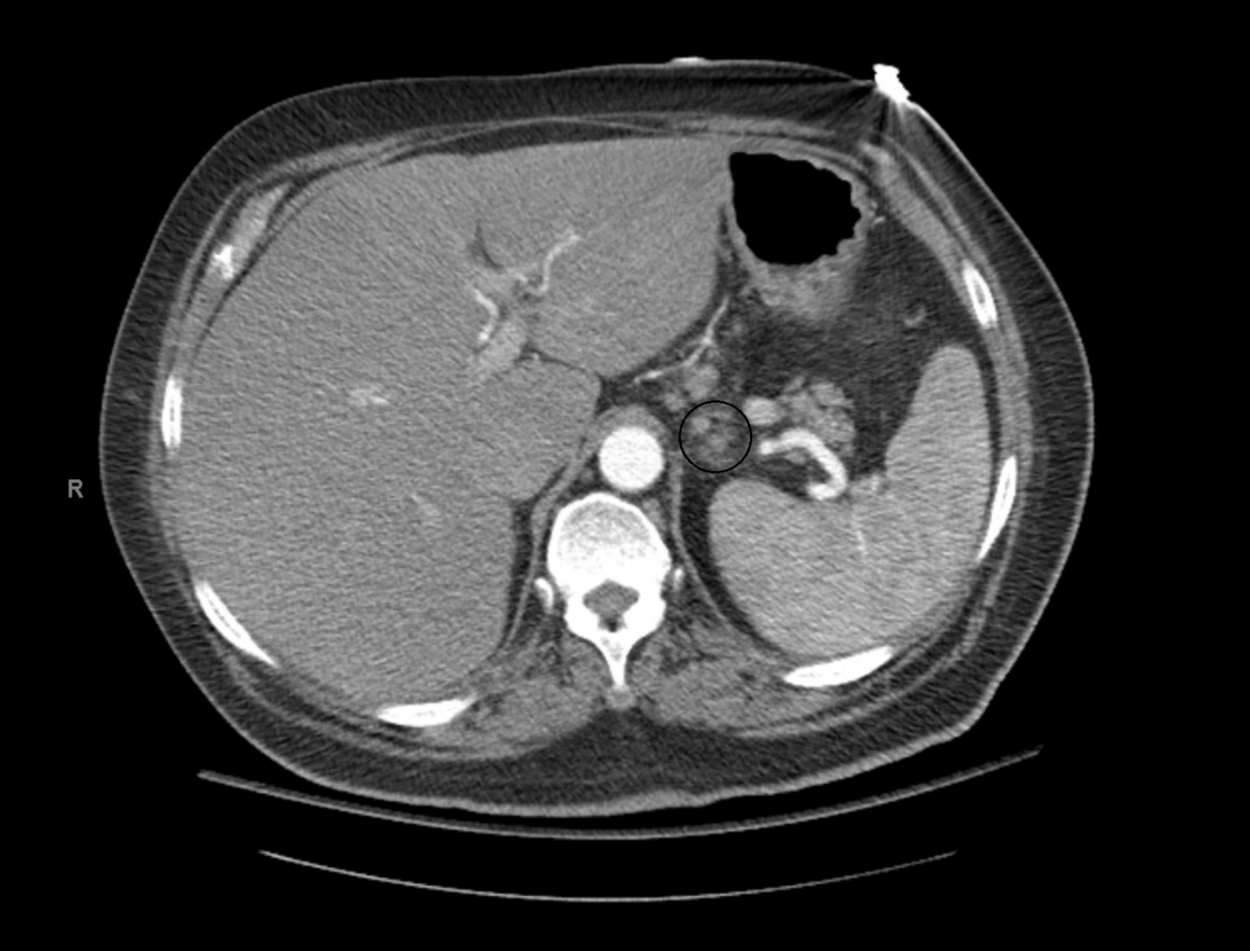
Multiple prominent gastrohepatic and portacaval lymph nodes

**Figure 2:**
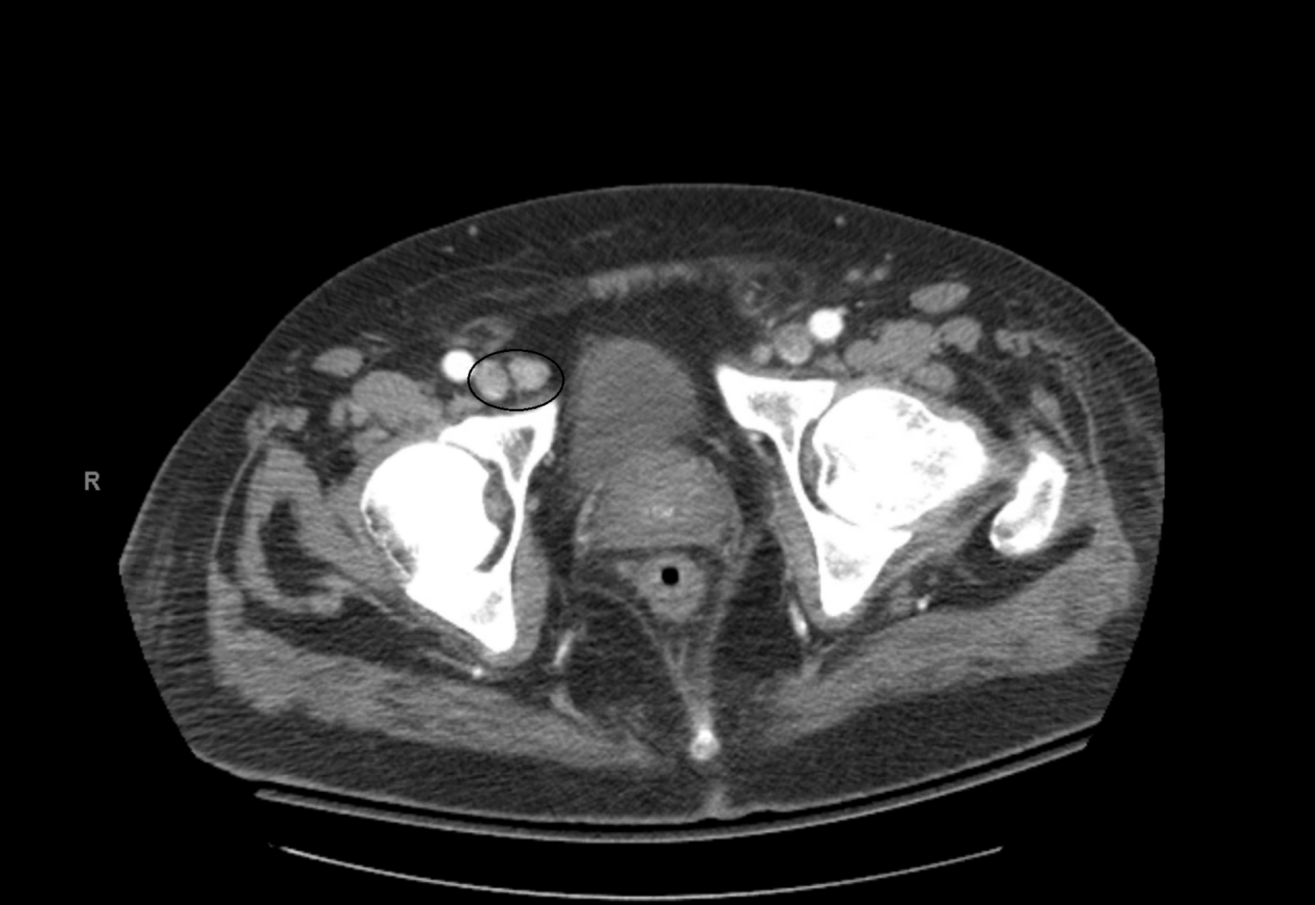
Enlarged right external iliac lymph node measuring up to 1.3 cm

A computed tomography (CT) scan of the abdomen with intravenous contrast showed a mildly enlarged periaortic, right external iliac lymph nodes, in addition to multiple prominent gastrohepatic and portacaval lymph nodes (Figures 1-2).

The patient initially responded appropriately to two units of packed red blood cells transfusion with hemoglobin improving to 7.8 g/dl. However, he later developed multiple episodes of melena and hematochezia causing acute blood loss anemia with hemoglobin drop to the 6.1 g/dl requiring two more units of packed red blood cell transfusions. He eventually developed an episode of massive upper gastrointestinal bleeding, associated with tachycardia (up to 115 beats/minute) and a drop in his blood pressure to 103/61 mmHg. Due to persistent bleeding and clinical instability, he was transferred to the intensive care unit for resuscitation and management of hemorrhagic shock. He was started on intravenous crystalloid fluid resuscitation, in addition to an intravenous infusion of pantoprazole of 8 mg/hour. 

**Figure 3:**
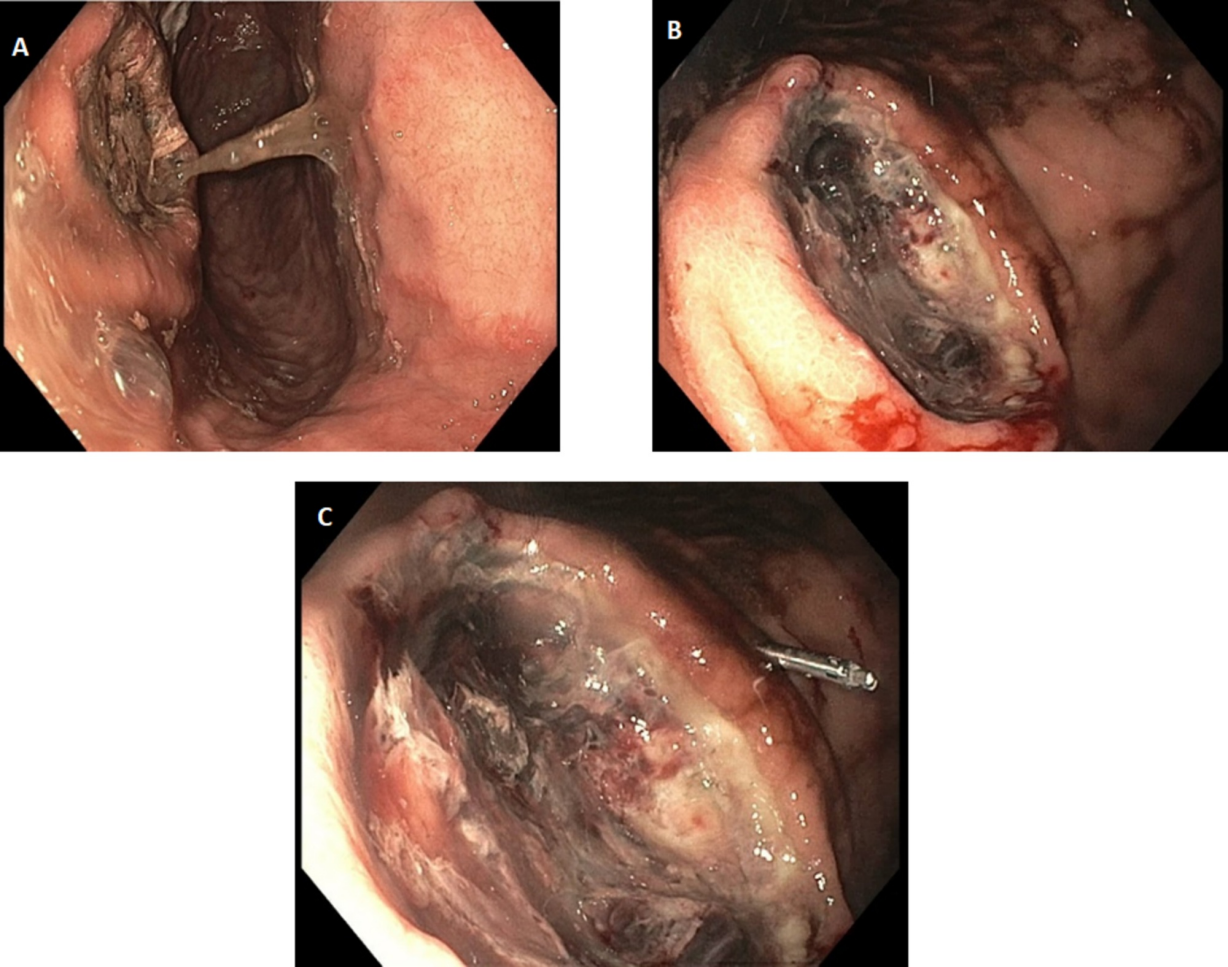
A) Non-bleeding erosions in the lower third of the esophagus; B,C) multiple non-bleeding gastric ulcers with a flat pigmented spot (Forrest class IIC)

After adequate resuscitation, an esophagogastroduodenoscopy showed non-bleeding erosions in the lower third of the esophagus, and multiple non-bleeding gastric ulcers with a flat pigmented spot (Forrest class IIC) (Figure 3). Biopsies were taken from the two largest ulcers with cold biopsy forceps for histology. Colonoscopy showed old clotted blood in the entire examined colon. 

The patient continued to have hematemesis associated with hypotension; a second esophagogastroduodenoscopy was performed a day following the first one and showed clotted blood in the gastric fundus, a large, cratered gastric body ulcer with an adherent clot (Forrest IIB). Most of the clot was removed without bleeding.

Due to persistent overt gastrointestinal bleeding and hypotension, a computed tomography angiogram showed the right gastroepiploic artery coursing through the large ulcer bed. To prevent subsequent bleeding, a selective embolization was performed. 

**Figure 4:**
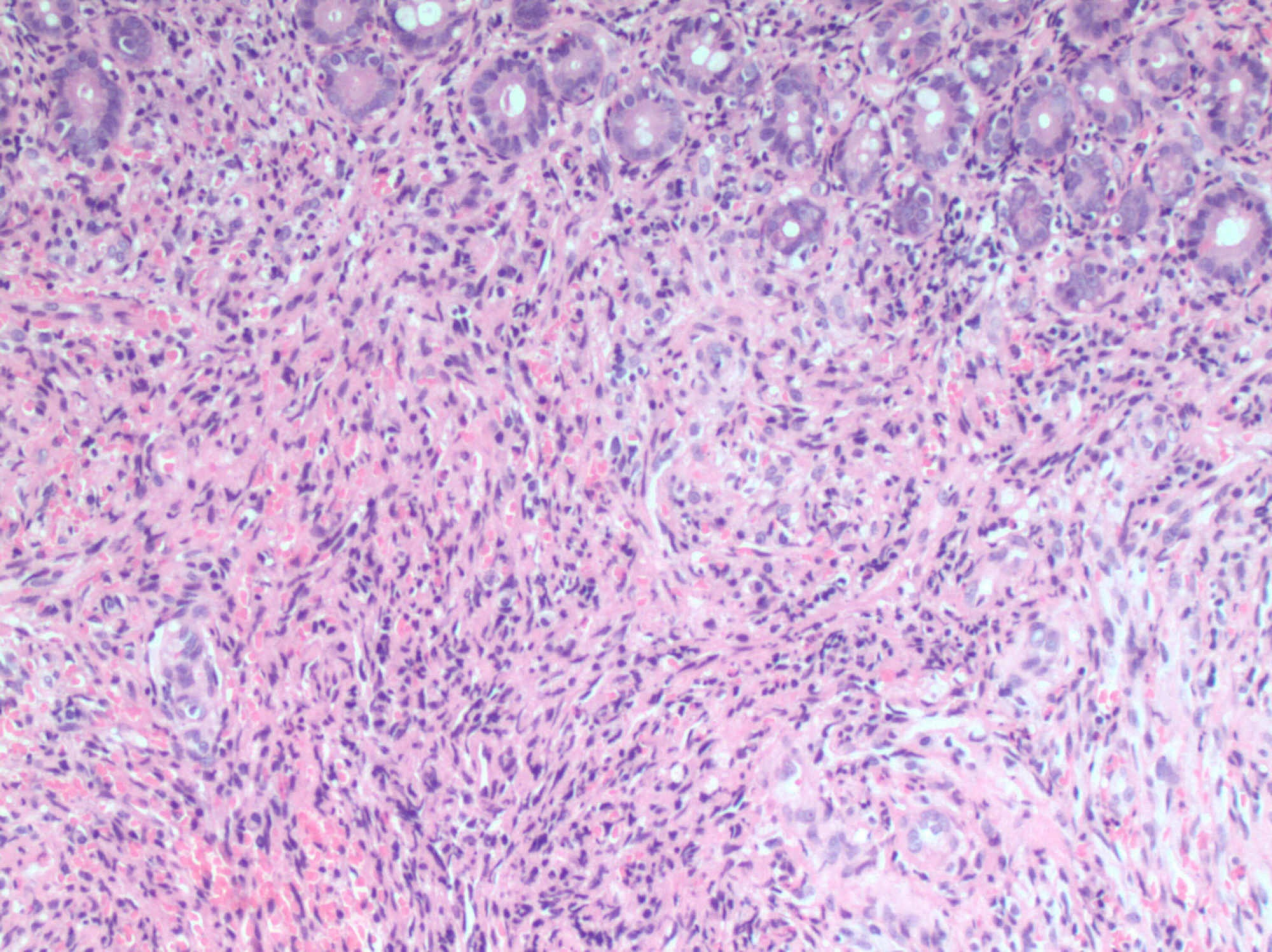
Duodenal mucosa demonstrates proliferating spindle cells forming slit-like vascular channels within the lamina propria

Histologic sections of the duodenal mucosa demonstrated proliferating spindle cells forming slit-like vascular channels within the lamina propria (Figure 4). Human herpes virus 8 immunostaining was positive. This was consistent with KS.

The patient remained hemodynamically stable after the embolization procedure, hemoglobin remained stable and no further episodes of gastrointestinal bleeding were noted. He was discharged on antiretroviral therapy with outpatient follow-up.

## Discussion

KS is classified into four major groups: classic, endemic, iatrogenic, and AIDS-related KS. Although the incidence of HIV infections has decreased dramatically since 2000, AIDS-related KS is still not uncommon [4]. KS is considered an AIDS defining illness. It is more common among homosexual as well as bisexual men [5]. The most common and first organ to be involved is the skin [6,7]. Visceral involvement, even without cutaneous manifestations, may occur [8].

The treatment of KS requires staging of the disease [9]. Antiretroviral therapy remains the first-line treatment, where studies have shown that using antiretroviral therapy in KS is associated with improved mortality and outcomes [10]. Other treatment options are available ranging from local intralesional chemotherapy to systemic chemotherapy depending on the disease stage and severity [11,12].

KS of the gastrointestinal tract is usually diagnosed by endoscopy and biopsies. Adequate tissue sampling is required, due to the high rate of false negative results, which can be as high as 77% [13]. KS lesions are hypervascular and tend to be submucosal, which makes tissue sampling very challenging. Biopsy of these lesions can result in significant gastrointestinal bleeding, which can be life-threatening, similar to what happened in our case.

In AIDS patients with a suspected gastrointestinal KS, we suggest that endoscopy is performed with high caution [14]. It is also recommended that such a procedure is performed in an advanced and monitored setting, where further therapies can be provided if needed, such as radiology guided embolization, in case of massive bleeding.

## Conclusions

As we mentioned above, KS is a defining disease of AIDS, it is not uncommon for the disease to involve the gastrointestinal tract. We have described a case where the disease caused severe life-threatening bleeding that warranted embolization of the involved artery. In cases where KS of the gastrointestinal tract is suspected, a very cautious approach is necessary to avoid complications. The use of antiretroviral therapy remains the first and most important treatment of the disease.
